# Human Papillomavirus Vaccination Coverage Among Adolescent Girls Aged 13–17 Years — U.S.-Affiliated Pacific Islands, 2013–2023

**DOI:** 10.15585/mmwr.mm7333a2

**Published:** 2024-08-22

**Authors:** Ashley Tippins, Glodi Mutamba, E.M. Boyd, Kelsey C. Coy, Jennifer L. Kriss

**Affiliations:** ^1^Immunization Services Division, National Center for Immunization and Respiratory Diseases, CDC; ^2^Eagle Health Analytics, San Antonio, Texas.

**Summary**

**What is already known about this topic?**

Cervical cancer is the fourth most common cancer among women worldwide, and the World Health Organization Western Pacific Region, where the U.S.-affiliated Pacific Islands (USAPI) are located, accounts for one quarter of all estimated cases. Human papillomavirus (HPV) vaccines prevent most cervical cancers and are recommended for girls at age 11–12 years.

**What is added by this report?**

This is the first comprehensive report of trends in HPV vaccination coverage among adolescent girls since the vaccines were introduced in USAPI jurisdictions. Coverage with HPV vaccine is on track to meet 2030 goals in two jurisdictions, but disparities need to be addressed.

**What are the implications for public health practice?**

Adolescent vaccination coverage assessment identifies progress toward regional goals. To target increased vaccine access and coverage, this assessment identifies populations and areas with low coverage.

## Abstract

Worldwide, cervical cancer is the fourth most common cancer among women, and the World Health Organization (WHO) Western Pacific Region, where the U.S.-affiliated Pacific Islands (USAPI) are located, accounts for one quarter of all estimated cases. Human papillomavirus (HPV) vaccines are recommended at age 11–12 years to prevent most cervical cancers. HPV vaccines were introduced across USAPI during 2007–2016, predominantly provided through school-located vaccination programs. Retrospective analysis using data from jurisdictional immunization information systems was used to estimate vaccination coverage among adolescent girls as of the last day of each calendar year during 2013–2023. This analysis measured progress toward the WHO 2030 vaccination coverage goal of ≥90% completion of the HPV vaccination series among girls by age 15 years. As of December 2023, initiation of the HPV vaccination series among adolescent girls aged 13–17 years ranged from 58.0% in Palau to 97.2% in the Northern Mariana Islands, and HPV vaccination series completion coverage ranged from 43.4% in Palau to 91.8% in the Northern Mariana Islands. HPV vaccination series completion coverage is >90% in the Northern Mariana Islands and is on track to meet WHO goals by 2030 in American Samoa. Assessment of adolescent vaccination coverage can help immunization programs monitor progress toward regional goals and identify populations and areas with low coverage. Implementing evidence-based strategies to increase vaccine access and coverage would benefit jurisdictions with lagging coverage.

## Introduction

Worldwide, cervical cancer is the fourth most common cancer among women, and the World Health Organization (WHO) Western Pacific region* accounts for one quarter of all estimated cases (*1*); the age-standardized rate of cervical cancer in the Marshall Islands (74 per 100,000 women) is the highest in the world (*2*). Nearly all cervical cancers are caused by human papillomaviruses (HPV). HPV vaccines, which have been licensed for use since 2006, are estimated to have the potential to prevent approximately 75% of all cervical cancers (*3*). CDC recommends HPV vaccination for both boys and girls at age 11–12 years.^†^ However, to assess progress toward reaching vaccination goals in the 2020 WHO Global Strategy to Accelerate the Elimination of Cervical Cancer as a Public Health Problem,^§^ this report focuses on HPV vaccination coverage among adolescent girls. The WHO strategy recommends that HPV vaccines be included in all national immunization programs; the goal is for ≥90% of girls to complete the HPV vaccination series by age 15 years, by 2030 (*4*).

HPV vaccines were introduced across the U.S.-affiliated Pacific Islands (USAPI)^¶^ during 2007–2016,** predominantly provided through school-located vaccination programs.^††^ Assessment of vaccination coverage among adolescent girls can help immunization programs monitor progress toward regional goals and identify populations and areas with low coverage. These data can be used to guide evidence-based interventions, adapted to the local context, to improve vaccination coverage. This report describes annual HPV vaccination coverage among adolescent girls in five of the six USAPI^§§^ jurisdictions during 2013–2023.

## Methods

### Data Sources and Inclusion and Exclusion Criteria

Patient-level data from jurisdictional immunization information systems (IISs) were aggregated at the jurisdiction level for this retrospective analysis. Persons were included in the denominator for annual analyses if they 1) were adolescent girls aged 13–17 years as of January 1 of the assessment year, 2) had an active patient status^¶¶^ in the IIS through the end of the assessment year, and 3) had received any vaccine within the most recent 5 years. Exclusion criteria consistent with the Modeling of Immunization Registry Operations Work Group managing active patient status guidance was retrospectively applied to mitigate IIS denominator inflation (*5*). Patients were excluded from all analyses if they had zero vaccine doses recorded in the IIS or if the last vaccination date recorded in the IIS was before January 1, 2006.

### Estimation of HPV Vaccination Coverage

Retrospective point-in-time analysis (i.e., coverage as of a specific date) was used to estimate vaccination coverage as of December 31 of each year during 2013–2023. All HPV vaccine doses received as of the end of the assessment year were included in coverage estimates. Vaccination coverage indicators included receipt of ≥1 HPV vaccine dose and HPV vaccination series completion status.*** Completion of the HPV vaccination series is defined as receipt of ≥3 HPV vaccine doses, or receipt of 2 doses if the series was initiated at age <15 years, and if ≥5 months minus 4 days have elapsed between receipt of the first and second dose.

HPV vaccination series dropout was measured as the proportion of adolescents who had not completed the HPV vaccination series by the end of the assessment year, among those who received the first dose. SAS software (version 9.4; SAS Institute) was used to conduct all analyses. This activity was reviewed by CDC, deemed not research, and was conducted consistent with applicable federal law and CDC policy.^†††^

## Results

### Jurisdictional HPV Vaccination Coverage Among Adolescent Girls Aged 13–17 Years

Coverage with ≥1 HPV vaccine dose and HPV vaccination series completion status varied by jurisdiction (Table). As of December 2023, coverage with ≥1 HPV dose among adolescent girls aged 13–17 years ranged from 58.0% in Palau to 97.2% in the Northern Mariana Islands. HPV vaccination series completion coverage ranged from 43.4% in Palau to 91.8% in the Northern Mariana Islands. As of 2023, the Northern Mariana Islands is the only jurisdiction to have already met the WHO 2030 HPV 90% vaccination coverage goal.

**TABLE:** Human papillomavirus vaccination coverage among adolescent girls aged 13–17 years, by jurisdiction — U.S.-affiliated Pacific Islands,* 2013–2023

Jurisdiction/Year	Population,^†^ no.	Coverage, %		
		Received ≥1 HPV vaccine dose	HPV vaccination series completion^§^	HPV vaccination series dropout^¶^
**American Samoa**				
2013	3,785	23.0	4.9	78.8
2014	3,888	36.5	12.9	64.7
2015	3,937	48.1	25.2	47.6
2016	3,608	59.6	34.6	42.0
2017	3,580	62.8	36.0	42.7
2018	3,578	67.7	39.1	42.2
2019	3,492	79.4	50.0	37.1
2020	3,291	86.0	61.0	29.0
2021	3,194	89.0	67.6	24.0
2022	3,027	92.6	78.0	15.8
2023	2,921	95.7	82.8	13.4
**Northern Mariana Islands**				
2013	3,142	62.1	44.2	28.8
2014	3,155	59.4	43.0	27.5
2015	3,053	57.5	40.6	29.3
2016	2,904	60.5	44.2	26.8
2017	2,733	79.8	56.1	29.7
2018	2,673	85.3	72.1	15.5
2019	2,591	88.5	79.7	9.9
2020	2,476	92.4	87.0	5.9
2021	2,511	92.7	87.6	5.5
2022	2,314	95.2	90.2	5.3
2023	2,289	97.2	91.8	5.6
**Federated States of Micronesia**				
2013	6,807	17.3	9.4	45.8
2014	6,914	17.5	9.7	44.2
2015	6,854	18.0	10.2	43.1
2016	6,853	26.2	12.6	52.1
2017	6,769	29.6	16.0	45.9
2018	6,800	32.3	19.5	39.7
2019	6,789	35.1	23.6	32.8
2020	5,956	45.6	33.9	25.5
2021	5,766	52.6	40.9	22.2
2022	5,539	55.9	45.6	18.5
2023	5,507	59.5	48.4	18.5
**Marshall Islands**				
2013	3,358	27.2	13.6	49.8
2014	3,400	26.8	13.8	48.4
2015	3,373	26.0	13.0	50.2
2016	3,368	32.2	16.4	49.0
2017	3,402	39.7	22.3	43.9
2018	3,475	47.7	30.7	35.6
2019	3,528	57.2	39.7	30.6
2020	3,518	63.2	45.3	28.3
2021	3,476	66.5	48.1	27.7
2022	3,231	70.2	52.2	25.6
2023	2,981	71.4	53.6	24.9
**Palau**				
2013	893	10.3	8.1	21.7
2014	786	18.8	12.2	35.1
2015	721	30.1	19.6	35.0
2016	673	41.9	27.8	33.7
2017	683	54.0	35.4	34.4
2018	650	62.6	45.8	26.8
2019	647	69.7	54.7	21.5
2020	630	71.6	59.0	17.5
2021	639	69.3	57.4	17.2
2022	623	64.2	52.2	18.8
2023	629	58.0	43.4	25.2

**Abbreviations**: HPV = human papillomavirus; IIS = immunization information system.

* Jurisdictions in this report include American Samoa, Northern Mariana Islands, Federated States of Micronesia, Marshall Islands, and Palau. Vaccination coverage among adolescents in Guam has been assessed via the National Immunization Survey since 2013; IIS-based coverage assessment was not conducted for Guam. Information on adolescent vaccination coverage in Guam is available at https://www.cdc.gov/vaccines/imz-managers/coverage/teenvaxview/data-reports/index.html.

^†^ Total number of adolescent girls aged 13–17 years with an active patient status in the IIS, ≥1 dose of any vaccine ever recorded in the IIS, and ≥1 dose of any vaccine recorded in the IIS within 5 years of the assessment year.

**^§^** In December 2016, the HPV vaccination recommendations changed from a 3-dose series for all to a 2-dose series among children and adolescents who initiate the vaccination series before age 15 years. Completion of the HPV vaccination series is defined as receipt of ≥3 HPV vaccine doses, or receipt of 2 doses if the series is initiated at age <15 years, and ≥5 months minus 4 days have elapsed between the first and second dose. This measure was applied retrospectively for all years 2013–2023. https://www.cdc.gov/vaccines/hcp/imz-schedules/child-adolescent-notes.html#note-hpv

**^¶^** The percentage of adolescents who started the HPV vaccination series but did not complete it as of the end of the assessment year.

### Trends in HPV Vaccination Coverage Among Adolescent Girls, 2013–2023

**FIGURE 1:**
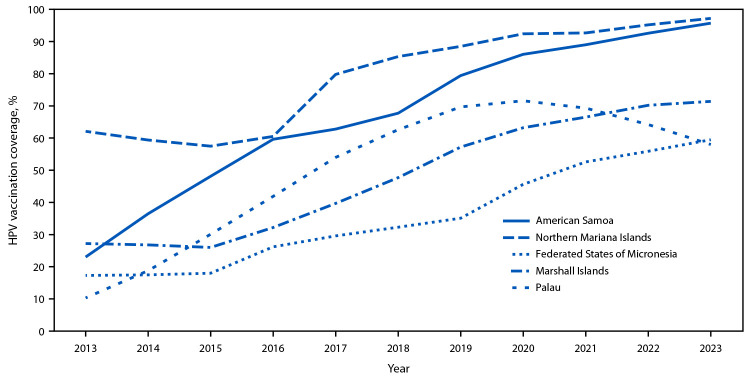
Trends in ≥1-dose human papillomavirus vaccination coverage among adolescent girls aged 13–17 years, by jurisdiction — U.S.-affiliated Pacific Islands,* 2013–2023 **Abbreviation:** HPV = human papillomavirus. * Jurisdictions include American Samoa, Northern Mariana Islands, Federated States of Micronesia, Marshall Islands, and Palau. Vaccination coverage among adolescents in Guam has been assessed via the National Immunization Survey since 2013; immunization information system–based coverage assessment was not conducted for Guam. https://www.cdc.gov/vaccines/imz-managers/coverage/teenvaxview/data-reports/index.html

**FIGURE 2:**
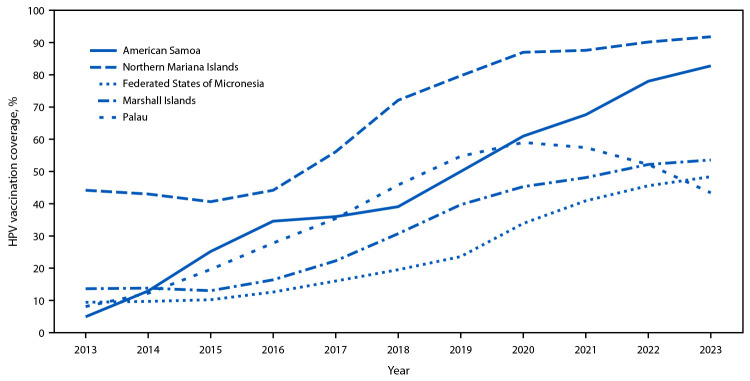
Trends in human papillomavirus vaccination series completion* coverage among adolescent girls aged 13–17 years, by jurisdiction**^^†^^** — U.S.-affiliated Pacific Islands, 2013–2023 **Abbreviation:** HPV = human papillomavirus. * In December 2016, the HPV vaccination recommendations changed from a 3-dose series for all to a 2-dose series among children and adolescents who initiate the vaccination series before age 15 years. Completion of the HPV vaccination series is defined as receipt of ≥3 HPV vaccine doses, or receipt of 2 doses if the series is initiated at age <15 years, and ≥5 months minus 4 days have elapsed between the first and second dose. This measure was applied retrospectively for all years 2013–2023. https://www.cdc.gov/vaccines/hcp/imz-schedules/child-adolescent-notes.html#note-hpv ^†^ Jurisdictions include American Samoa, Northern Mariana Islands, Federated States of Micronesia, Marshall Islands, and Palau. Vaccination coverage among adolescents in Guam has been assessed via the National Immunization Survey since 2013; immunization information system–based coverage assessment was not conducted for Guam. https://www.cdc.gov/vaccines/imz-managers/coverage/teenvaxview/data-reports/index.html

During 2013–2023, coverage with ≥1 HPV vaccine dose increased by 35.2–72.8 percentage points across jurisdictions (Figure 1), and HPV vaccination series completion coverage increased by 35.3–72.9 percentage points (Figure 2). The percentage of adolescent girls who had received ≥1 HPV vaccine dose and who completed the vaccination series increased each year from 2013 to 2023 in all jurisdictions except Palau, where ≥1-dose coverage and HPV vaccination series completion coverage peaked in 2020 at 71.6% and 59.0%, respectively, and have since declined to 58.0% and 43.4%, respectively, in 2023 (Table). In American Samoa, HPV vaccination series completion coverage increased from 78.0% to 82.8% (4.8 percentage points) from 2022 to 2023. If coverage continues to increase at the same rate, American Samoa will meet the WHO 2030 ≥90% HPV vaccination series completion coverage goal by 2025.

HPV vaccination series dropout varied across jurisdictions and years; during 2013–2023, dropout decreased in all jurisdictions except in Palau, where it increased from a low of 17.2% in 2021 to a high of 25.2% in 2023 (Table). Dropout was lowest in the Northern Mariana Islands, where only 5.6% of adolescent girls aged 13–17 years who initiated the HPV vaccination series had not completed it in 2023.

## Discussion

HPV vaccines are a critical public health tool to prevent most cervical cancers. HPV vaccination coverage has increased markedly in USAPI since the vaccination programs commenced, and HPV vaccination series completion coverage in the Northern Mariana Islands currently exceeds the ≥90% WHO 2030 goal. If the current coverage trends continue, American Samoa will also be on track to meet the WHO 2030 coverage goal. The 2023 rates of initiation of the HPV vaccination series among adolescent girls aged 13–17 years in American Samoa (95.7%) and the Northern Mariana Islands (97.2%) are higher than those in the three freely associated USAPI jurisdictions (Federated States of Micronesia, Marshall Islands, and Palau) (range = 58.0%–71.4%).

The differences in coverage among the USAPI jurisdictions might be attributed, at least in part, to differences in access to the vaccine. Both American Samoa and the Northern Mariana Islands have offered the vaccine through a mix of school-located vaccination programs as well as in public health clinics. These two jurisdictions receive vaccination program funding and vaccine supply through the Section 317 Immunization Program and the U.S. Vaccines for Children (VFC) program. The three freely associated jurisdictions are not eligible to receive VFC funding and thus have a more limited vaccine supply; therefore, these jurisdictions have not consistently been able to offer HPV vaccine in clinics or other locations outside the school setting.

The school-located HPV vaccination program is an evidence-based intervention to increase HPV vaccination coverage, particularly in low- and middle-income settings; however, jurisdiction-level coverage is constrained when the vaccine is only available in the school setting (*6*). For example, secondary school enrollment^§§§^ among girls is approximately 66% in Federated States of Micronesia, 83% in Marshall Islands, and 80% in Palau, compared with approximately 97% in American Samoa and the Northern Mariana Islands (*7*–*9*). Strategies to reach out-of-school adolescent girls are needed to improve vaccination coverage in these settings. Providing vaccine access to girls who are not enrolled in school is also an important health equity consideration. Some research suggests that girls who drop out of school are more likely to contract sexually transmitted infections, such as HPV, than are those who remain in school (*10*).

In addition to challenges associated with accessing adolescent girls who are not in school, school-based HPV vaccination programs in some areas might have been suspended while schools were closed during the COVID-19 pandemic. The decline in coverage after 2020 in Palau might be evidence of the pandemic’s impact because coverage was trending up among girls who reached the target vaccination age of 11–12 years before 2020, compared with girls who reached age 11–12 years in 2020 and later. More research is needed to assess the underlying reasons for the lower coverage in the freely associated USAPI and to design and implement evidence-based interventions to improve vaccination outcomes adapted to the local context. Specific strategies might be needed to increase vaccination coverage among populations that have recently experienced larger declines in coverage, including those who would have been within the recommended age for vaccination during the pandemic.

### Limitations

The findings in this report are subject to at least three limitations. First, accuracy of coverage estimates in this assessment is dependent upon completeness and accuracy of jurisdictional IIS data. Working with the jurisdictions, CDC has found high levels of completeness and accuracy of vaccination data (i.e., consistency in recorded dose dates and product types between paper and IIS records) across the five USAPI IISs included in this assessment through evaluations conducted since 2016. However, IIS data completeness before 2016 has not been evaluated. Second, the active patient population size could be inflated in IISs compared with census estimates because of difficulties tracking out-migration and deaths, which can lead to an underestimation of vaccination coverage. However, recent U.S. Census Bureau data were not available for denominator estimation for all jurisdictions included in this assessment. For this reason, exclusion criteria consistent with the Modeling of Immunization Registry Operations Work Group managing active patient status guidance were applied to retrospectively classify likely active patient status to patients in the IIS for each assessment year. Finally, vaccination coverage for Guam is assessed via the National Immunization Survey and was not included in this analysis. Differences in vaccination coverage estimation methods might mean that results are not directly comparable with IIS-based estimates for the other USAPI presented in this report.

### Implications for Public Health Practice

Only two of the five USAPI have met or are on track to meet the WHO 2030 goal of ≥90% completion of the HPV vaccination series among girls by age 15 years. Identifying and implementing evidence-based strategies to increase vaccine access and coverage would benefit jurisdictions with lagging coverage. The USAPI immunization programs partner with various international governmental, nongovernmental, and academic organizations on immunization and comprehensive cancer control initiatives. Vaccination coverage data can support development of their activities by providing performance indicators and data for modeling health outcomes related to HPV vaccination, promoting health equity, and attaining the WHO 2030 goal of 90% HPV vaccination series completion coverage.

## Footnotes

* WHO member countries are grouped into six regions: Africa, Americas, Eastern Mediterranean, Europe, South-East Asia, and Western Pacific. The Western Pacific Region consists of 37 countries and areas, including the six U.S.-affiliated Pacific Islands. https://www.who.int/westernpacific/about/where-we-work

†
https://www.cdc.gov/vaccines/imz-schedules/adolescent-easyread.html

§ Three of the six USAPI jurisdictions offer the vaccine to girls (American Samoa, Guam, and the Northern Mariana Islands), and three offer the vaccine to both boys and girls (Federated States of Micronesia, Marshall Islands, and Palau). Coverage among adolescent girls is included in this report for consistency across jurisdictions and to assess progress toward WHO goals, which include vaccination goals only for girls. https://www.cdc.gov/vaccines/hcp/imz-schedules/child-adolescent-notes.html#note-hpv

¶ USAPI comprise three U.S. territories (American Samoa, Guam, and the Northern Mariana Islands) and three freely associated nations (Federated States of Micronesia, Marshall Islands, and Palau). All jurisdictions receive Section 317 Immunization Program funding, which is a discretionary program funded by the U.S. Congress to purchase vaccines and support immunization infrastructure. The U.S. territories (American Samoa and Northern Mariana Islands) also receive Vaccines for Children (VFC) funding; VFC is an entitlement program for children aged ≤18 years that provides vaccines at no cost to VFC program–eligible children through public and private health care providers that are enrolled in the VFC program.

** Implementation of the HPV vaccine program varied by jurisdiction. Northern Mariana Islands implemented the program in 2007, Palau in 2008, Marshall Islands in 2009, and American Samoa in 2011. Federated States of Micronesia implemented the program in three states (Kosrae, Pohnpei, and Yap) in 2009, and the fourth state (Chuuk) in 2016.

†† American Samoa provided a mixed clinic- and school-located HPV program during 2011–2018. The school-located program ended in 2018.

§§ Jurisdictions in this report include American Samoa, Northern Mariana Islands, Federated States of Micronesia, Marshall Islands, and Palau. Vaccination coverage among adolescents in Guam has been assessed via the National Immunization Survey since 2013; immunization information system (IIS)–based coverage assessment was not conducted for Guam. Information on adolescent vaccination coverage in Guam is available at https://www.cdc.gov/vaccines/imz-managers/coverage/teenvaxview/data-reports/index.html.

¶¶ Patient active or inactive status in the IIS establishes a classification of individual patients within a health care organization. Health care providers are responsible for vaccinating patients with an “active” status within their clinic population or geographic catchment area. Patient status is changed to “inactive” when the patient changes providers, moves, or is lost to follow-up or “deceased” if patient death is confirmed through manual review or system linkage with vital statistics or other health records. https://repository.immregistries.org/files/resources/5835adc2dad8d/mirow_pais_mini-guide.pdf

*** In 2016, the HPV vaccine recommendations changed from a 3-dose series for all to a 2-dose series among children and adolescents who initiate the vaccination series before age 15 years. Completion of the HPV vaccination series is defined as receipt of ≥3 HPV vaccine doses or receipt of 2 doses if the series is initiated at age <15 years, and ≥5 months minus 4 days have elapsed between the first and second dose. This measure was applied retrospectively for all years 2013–2023. https://www.cdc.gov/vaccines/hcp/imz-schedules/child-adolescent-notes.html#note-hpv

††† 45 C.F.R. part 46, 21 C.F.R. part 56; 42 U.S.C. Sect. 241(d); 5 U.S.C. Sect. 552a; 44 U.S.C. Sect. 3501 et seq.

§§§ Gross enrollment ratio, measured as the ratio of total enrollment, regardless of age, to the population of the age group that officially corresponds to the level of education shown. Data for American Samoa and the Northern Mariana Islands are available from the U.S. Census Bureau. https://data.census.gov/table?q=school%20enrollment%20and%20sex%20american%20samoa; https://data.census.gov/table?q=school%20enrollment%20and%20sex%20commonwealth%20of%20the%20northern%20mariana%20islands
